# Quantitative unique continuation for the heat equations with inverse square potential

**DOI:** 10.1186/s13660-018-1907-4

**Published:** 2018-11-14

**Authors:** Guojie Zheng, Keqiang Li, Yuanyuan Zhang

**Affiliations:** 1 College of Mathematics and Information Science, Henan Normal University, Xinxiang, P.R. China; 2 Xinxiang No. 7 Middle School, Xinxiang, P.R. China

**Keywords:** 35K67, Heat equations, Singular potential, Unique continuation, Frequency function

## Abstract

In this paper, we investigate the unique continuation properties for multi-dimensional heat equations with inverse square potential in a bounded convex domain Ω of $\mathbb{R}^{d}$. We establish observation estimates for solutions of equations. Our result shows that the value of the solutions can be determined uniquely by their value on an open subset *ω* of Ω at any given positive time *L*.

## Introduction

In this paper, we consider the quantitative unique continuation for multi-dimensional heat equations with a singular potential term. The heat equations studied in this article are described by

1.1 $$ \textstyle\begin{cases} \partial_{t}\varphi(x,t)-\triangle\varphi(x,t)-V(x)\varphi(x,t)=0 & \text{in } \Omega\times(0,L], \\ \varphi(x,t)=0 & \text{on }\partial\Omega\times(0,L], \\ \varphi(x,0)=\varphi_{0}(x) & \text{in }\Omega, \end{cases} $$

where *L* is a positive number, $\Omega\subset \mathbb{R}^{d}$ ($d\geq3$) is a convex and bounded domain with smooth boundary *∂*Ω and $x=0\in\Omega$. The potential function is

1.2 $$ V(x)=\frac{\mu}{|x|^{2}}, \quad \mu< \mu_{*}=\frac{(d-2)^{2}}{4}. $$

The well-posedness theory of these equations have mainly been studied in recent years. For the existence and other properties of solutions to equation (1.1), we refer to [2, 3, 7, 13, 19]. In particular, in [3], authors proved that if a non-negative initial value $\varphi_{0}\in L^{2}(\Omega)$ is prescribed, then there exists a unique global weak solution for equation (1.1) under assumption (1.2), but as $\mu>\mu_{*}$, the local solution may not exist. In [19], the well-posedness of equation (1.1) without the sign restriction for the solution is thoroughly discussed. In summary, for any initial value $\varphi_{0}\in L^{2}(\Omega)$, there exists a unique solution $\varphi\in C([0,T];L^{2}(\Omega))\cap L^{2}(0,T;H_{0}^{1}(\Omega ))$ for equation (1.1) with (1.2). Throughout the paper, we use $\|\cdot\|$ and $\langle \cdot,\cdot\rangle$ to denote the usual norm and the inner product in the space $L^{2}(\Omega)$, respectively. Besides, variables *x* and *t* for functions of $(x, t)$ and variable *x* for functions of *x* will be omitted, provided that it is not going to cause any confusion.

The main results are presented as follows.

### Theorem 1.1

*Suppose that* *ω* *is a non*-*empty open subset of* Ω, $0\in \omega$, *and* $\varphi_{0}\in L^{2}(\Omega)$. *Then there exist two positive numbers* $\alpha=\alpha(\Omega,\omega)$, $C=C(\Omega,\omega)$ *such that*, *for each* $L>0$,

1.3 $$ \int_{\Omega}\bigl|\varphi(x,L)\bigr|^{2}\,dx\leq Ce^{\frac{C}{L}} \biggl( \int_{\Omega}|\varphi_{0}|^{2}\,dx \biggr)^{1-\alpha}\biggl( \int_{\omega}\bigl|\varphi(x,L)\bigr|^{2}\,dx\biggr)^{\alpha}. $$

*Moreover*, *if* $\varphi_{0}\neq0$, *then*

1.4 $$ \|\varphi_{0}\|_{H^{-1}(\Omega)}^{2}\leq C\exp \biggl(\frac{C}{L}+ CL\frac{\|\varphi(x,0)\|_{L^{2}(\Omega)}^{2}}{\|\varphi(x,0)\| _{H^{-1}(\Omega)}^{2}} \biggr) \int_{\omega}\bigl\vert \varphi(L) \bigr\vert ^{2} \,dx. $$

### Remark 1.1

(i) The mathematical model (1.1) is a special case where potential term $V(x)=\lambda/|x|^{2}$. The singular potentials occur in many physical phenomena. In non-relativistic quantum mechanics, the harmonic oscillator and the Coulomb central potential are typical examples of such kind (see [12]). In particular, it can also be found in the study of quantum scattering theory (see [17]). Thus, it is very significant to study the properties of equation (1.1).
(ii) The constant *C* in (1.3) or (1.4) stands for a positive constant only depending on Ω and *ω*. Specifically, it depends on the size of *ω* and Ω, and the distance from *ω* to *∂*Ω.
(iii) These results demonstrate that solutions of (1.1) can be uniquely determined by its value on an open subset *ω*, which contains zero, at any given positive time *L*.

The study of unique continuation for the solutions of PDEs began at the beginning of the last century. It plays an important role in PDEs theory, inverse problems, and control theory. To the best of our knowledge, the first result for strong unique continuation of parabolic equations was derived in 1974 in [10]. In [10], the authors established the unique continuation for parabolic equations with time independent coefficients by the properties of eigenfunctions of the corresponding elliptic operator, and this approach cannot be applied to parabolic equations with time dependent coefficients. From 1980s, there have been more results of unique continuation for parabolic equations, and we refer the readers to [5, 8, 9, 11, 14–16] and rich references cited therein. In our paper, we mainly study this property for the heat equations with the inverse square potential. The main difficulty in proving Theorem 1.1 lies in the singular potential terms. This difficulty is overcome by setting up a new norm for $H_{0}^{1}(\Omega)$ in terms of the Hardy–Poincaré inequality. With the aid of the frequency function, we can obtain those quantitative estimates.

We organize this paper as follows: In Sect. 2, we give some preliminary results; Sect. 3 is devoted to the proof of Theorem 1.1.

## Preliminary results

We suppose that $\Omega\subset\mathbb{R}^{d}$ ($d \ge3$) is an open domain with a smooth boundary *∂*Ω and $0\in\Omega$. Let us first recall the well-known Hardy–Poincaré inequality that there exists a positive constant $C(\Omega)$, which only depends on Ω, such that

2.1 $$ \int_{\Omega} \biggl[ \bigl\vert \nabla v(x) \bigr\vert ^{2}-\mu_{*}\frac{v^{2}(x)}{|x|^{2}} \biggr]\,dx\geq C(\Omega) \int_{\Omega} v^{2}(x)\,dx, \quad \forall v\in H^{1}_{0}(\Omega), $$

where $\mu_{*}$ is provided in (1.2). The proof for inequality (2.1) can be found in [4, 13]. Furthermore, as $\mu<\mu_{*}$,

2.2 $$ \int_{\Omega} \biggl[ \bigl\vert \nabla v(x) \bigr\vert ^{2}-\mu\frac{v^{2}(x)}{ \vert x \vert ^{2}} \biggr]\,dx \ge C(\Omega) \biggl(1- \frac{\mu}{\mu_{*}} \biggr) \int_{\Omega} \bigl\vert \nabla v(x) \bigr\vert ^{2} \,dx+\frac{C(\Omega)\mu}{\mu_{*}} \int_{\Omega}v^{2}(x)\,dx. $$

By (2.2), we can equip $H_{0}^{1}(\Omega)$ with the following inner product:

2.3 $$ \langle f,g\rangle_{H_{0}^{1}(\Omega)}= \int_{\Omega} \bigl[\nabla f(x)\cdot\nabla g(x)-V(x)f(x)g(x) \bigr] \,dx, \quad \forall f,g\in H_{0}^{1}(\Omega), $$

and the norm $\|f\|_{H_{0}^{1}(\Omega)}=(\int_{\Omega}(|\nabla f|^{2}-V(x)v^{2})\,dx)^{\frac{1}{2}}$ is equivalent to the standard norm in $H_{0}^{1}(\Omega)$. Taking $L^{2}(\Omega)$ as a pivot space, we have the following compact embeddings (see [18]):

$$ H_{0}^{1}(\Omega) \hookrightarrow L^{2}(\Omega) \hookrightarrow H^{-1}(\Omega) $$

and

2.4 $$ \langle f,g\rangle_{H^{-1}(\Omega),H_{0}^{1}(\Omega)}=\langle f,g\rangle_{L^{2}(\Omega)}, \quad \forall f\in L^{2}(\Omega), g\in H_{0}^{1}( \Omega). $$

For each $\lambda>0$, we define the following weight function over $\mathbb{R}^{d}\times[0,L]$:

2.5 $$ G_{\lambda}(x,t)=\frac{1}{(L-t+\lambda)^{d/2}}e^{-\frac {|x|^{2}}{4(L-t+\lambda)}}. $$

Then, for each $t\in[0,L]$, we define the following three functions over the interval $[0, L]$:

2.6 $$\begin{aligned}& H_{\lambda}(t)= \int_{\Omega} \bigl\vert \varphi(x,t) \bigr\vert ^{2}G_{\lambda}(x,t)\,dx, \end{aligned}$$

2.7 $$\begin{aligned}& D_{\lambda}(t)= \int_{\Omega} \biggl[ \bigl\vert \nabla\varphi(x,t) \bigr\vert ^{2}-\frac {\mu \vert \varphi(x,t) \vert ^{2}}{|x|^{2}} \biggr]G_{\lambda}(x,t)\,dx, \end{aligned}$$

and

2.8 $$ N_{\lambda}(t)=\frac{2D_{\lambda}(t)}{H_{\lambda}(t)}, $$

where $\varphi(x,t)$ is the solution of equation (1.1). The function $N_{\lambda}(t)$ was first discussed in [1]. It was called frequency function (see also [5, 6], and [16]). In this article, we define a different frequency function based on the new norm of $H_{0}^{1}(\Omega)$. We always suppose $H_{\lambda}(t)\neq0$. Now, we will discuss the properties for the functions $G_{\lambda}(x,t)$.

### Lemma 2.1

*For each* $\lambda>0$, *the function* $G_{\lambda}$ *given in* (2.5) *has the following identities over* $\mathbb{R}^{d}\times[0,L]$:

2.9 $$\begin{aligned}& \partial_{t}G_{\lambda}(x,t)+\triangle G_{\lambda}(x,t)=0, \end{aligned}$$

2.10 $$\begin{aligned}& \nabla G_{\lambda}(x,t)=\frac{-x}{2(L-t+\lambda)}G_{\lambda}(x,t), \end{aligned}$$

2.11 $$\begin{aligned}& \partial_{i}^{2}G_{\lambda}(x,t)= \frac{-1}{2(L-t+\lambda)}G_{\lambda}(x,t)+\frac{|x_{i}|^{2}}{4(L-t+\lambda)^{2}}G_{\lambda}(x,t), \end{aligned}$$

*and for* $i\neq j$,

2.12 $$ \partial_{i}\partial_{j}G_{\lambda}(x,t)= \frac{x_{i}x_{j}}{4(L-t+\lambda )^{2}}G_{\lambda}(x,t). $$

Next, we will study the properties for derivatives of the functions $H_{\lambda}(t)$, $D_{\lambda}(t)$, and $N_{\lambda}(t)$ in the following lemmas.

### Lemma 2.2

*For any* $\lambda>0$, *the following identity holds*:

2.13 $$ H_{\lambda}'(t)=-2D_{\lambda}(t), $$

*and*

2.14 $$ H_{\lambda}'(t)=2 \int_{\Omega}\varphi\biggl(\partial_{t}\varphi-\nabla \varphi\frac{x}{2(L-t+\lambda)} \biggr)G_{\lambda}\,dx. $$

### Proof

By direct computation, we obtain

2.15 $$\begin{aligned} H_{\lambda}'(t) =&2 \int_{\Omega}\varphi\partial_{t}\varphi G_{\lambda} \,dx+ \int_{\Omega}|\varphi|^{2}\partial_{t} G_{\lambda}\,dx \\ =&2 \int_{\Omega}\varphi\partial_{t}\varphi G_{\lambda} \,dx- \int _{\Omega}|\varphi|^{2}\triangle G_{\lambda}\,dx \\ =&2 \int_{\Omega}\varphi(\partial_{t}\varphi-\triangle \varphi) G_{\lambda}\,dx-2 \int_{\Omega}|\nabla\varphi|^{2} G_{\lambda }\,dx \\ =&-2 \int_{\Omega}\biggl(\nabla|\varphi|^{2}- \frac{\mu\varphi^{2}}{|x|^{2}}\biggr) G_{\lambda}\,dx=-2D_{\lambda}(t). \end{aligned}$$

Second,

2.16 $$\begin{aligned} H_{\lambda}'(t) =&2 \int_{\Omega}\varphi\partial_{t}\varphi G_{\lambda} \,dx- \int_{\Omega}|\varphi|^{2}\triangle G_{\lambda }\,dx \\ =&2 \int_{\Omega}\varphi\partial_{t}\varphi G_{\lambda} \,dx+ \int _{\Omega}\nabla|\varphi|^{2} \nabla G_{\lambda} \,dx \\ =&2 \int_{\Omega}\varphi\biggl(\partial_{t}\varphi-\nabla \varphi\frac {x}{2(L-t+\lambda)} \biggr)G_{\lambda}\,dx. \end{aligned}$$

This completes the proof of this lemma. □

### Remark 2.1

By Lemma 2.2, we have

$$ D_{\lambda}(t) =- \int_{\Omega}\varphi\biggl(\partial_{t}\varphi-\nabla \varphi\frac {x}{2(L-t+\lambda)} \biggr)G_{\lambda}\,dx. $$

### Lemma 2.3

*For any* $\lambda>0$, *the following identity holds*:

2.17 $$ D_{\lambda}'(t) = -\theta-2 \int_{\Omega}\biggl(\partial_{t} \varphi- \frac{x}{2(L-t+\lambda )}\nabla\varphi\biggr)^{2}G_{\lambda}\,dx+ \frac{1}{L-t+\lambda}D_{\lambda}(t), $$

*where*

$$ \theta= \int_{\partial\Omega}|\nabla\varphi|^{2} \frac{\partial G_{\lambda}}{\partial\nu}\,d \sigma-2 \int_{\partial\Omega} \frac {\partial\varphi}{\partial\nu} (\nabla\varphi\nabla G_{\lambda })\,d\sigma\geq0. $$

*Here and in what follows*, *ν* *is the outward unit normal vector of the surface* *∂*Ω.

### Proof

By the fact $\varphi=0$ on *∂*Ω, we first derive that

2.18 $$\begin{aligned} D_{\lambda}'(t) =&2 \int_{\Omega}\nabla\varphi\nabla\partial_{t}\varphi G_{\lambda }\,dx- \int_{\Omega}\frac{2\mu\varphi\partial_{t} \varphi }{|x|^{2}}G_{\lambda}\,dx+ \int_{\Omega}\biggl[|\nabla\varphi|^{2}- \frac {\mu\varphi^{2}}{|x|^{2}} \biggr]\partial_{t} G_{\lambda}\,dx \\ =&2 \int_{\Omega}\operatorname{div}(\partial_{t}\varphi \nabla\varphi G_{\lambda })\,dx-2 \int_{\Omega}\partial_{t}\varphi \operatorname{div}( \nabla\varphi G_{\lambda})\,dx \\ &{}-2 \int_{\Omega}\frac{\mu\varphi\partial_{t} \varphi}{|x|^{2}}- \int _{\Omega}\biggl[|\nabla\varphi|^{2}- \frac{\mu\varphi^{2}}{|x|^{2}} \biggr]\triangle G_{\lambda}\,dx \\ =&-2 \int_{\Omega}\partial_{t}\varphi\triangle\varphi G_{\lambda }\,dx-2 \int_{\Omega}\partial_{t}\varphi\nabla\varphi\nabla G_{\lambda}\,dx \\ &{}-2 \int_{\Omega}\partial_{t} \varphi\frac{\mu\varphi }{|x|^{2}}G_{\lambda} \,dx- \int_{\Omega}\biggl[|\nabla\varphi|^{2}- \frac {\mu\varphi^{2}}{|x|^{2}} \biggr]\triangle G_{\lambda}\,dx \\ =&-2 \int_{\Omega}\partial_{t}\varphi\biggl(\triangle\varphi+ \frac{\mu \varphi}{|x|^{2}}\biggr) G_{\lambda}\,dx-2 \int_{\Omega}\partial_{t}\varphi \nabla\varphi \frac{-x}{2(L-t+\lambda)} G_{\lambda}\,dx \\ &{}- \int_{\Omega}\biggl[|\nabla\varphi|^{2}- \frac{\mu\varphi^{2}}{|x|^{2}} \biggr]\triangle G_{\lambda}\,dx \\ =&-2 \int_{\Omega}(\partial_{t}\varphi)^{2} G_{\lambda}\,dx-2 \int _{\Omega}\partial_{t}\varphi\nabla\varphi \frac{-x}{2(L-t+\lambda)} G_{\lambda}\,dx \\ &{}- \int_{\Omega}\biggl[|\nabla\varphi|^{2}- \frac{\mu \varphi^{2}}{|x|^{2}} \biggr]\triangle G_{\lambda}\,dx. \end{aligned}$$

Now, we deal with the last term in (2.18). In fact,

$$\begin{aligned} \int_{\Omega}|\nabla\varphi|^{2}\triangle G_{\lambda}\,dx =& \int _{\partial\Omega}|\nabla\varphi|^{2} \frac{\partial G_{\lambda }}{\partial\nu}\,d \sigma- \int_{\Omega}\nabla|\nabla\varphi|^{2}\nabla G_{\lambda}\,dx \\ =& \int_{\partial\Omega}|\nabla\varphi|^{2} \frac{\partial G_{\lambda}}{\partial\nu}\,d \sigma-2 \int_{\Omega}\nabla\varphi \nabla(\nabla\varphi\nabla G_{\lambda})\,dx \\ &{}+2\sum_{i=1}^{d} \int _{\Omega}\partial_{i}\varphi(\nabla\varphi \partial_{i}\nabla G_{\lambda})\,dx \\ =& \int_{\partial\Omega}|\nabla\varphi|^{2} \frac{\partial G_{\lambda}}{\partial\nu}\,d \sigma-2 \int_{\Omega}\operatorname{div}\bigl[\nabla\varphi (\nabla\varphi \nabla G_{\lambda})\bigr]\,dx \\ &{}+2 \int_{\Omega}\triangle\varphi(\nabla\varphi\nabla G_{\lambda }) \,dx+2\sum_{i=1}^{d} \int_{\Omega}\partial_{i}\varphi(\nabla\varphi \partial_{i}\nabla G_{\lambda}) \\ =& \int_{\partial\Omega}|\nabla\varphi|^{2} \frac{\partial G_{\lambda}}{\partial\nu}\,d \sigma-2 \int_{\partial\Omega} \frac {\partial\varphi}{\partial\nu} (\nabla\varphi\nabla G_{\lambda })\,d\sigma \\ &{}+2 \int_{\Omega}\triangle\varphi(\nabla\varphi\nabla G_{\lambda }) \,dx+2\sum_{i=1}^{d} \int_{\Omega}\partial_{i}\varphi(\nabla\varphi \partial_{i}\nabla G_{\lambda}). \end{aligned}$$

Thus,

2.19 $$\begin{aligned} \int_{\Omega}|\nabla\varphi|^{2}\triangle G_{\lambda}\,dx =&\theta +2 \int_{\Omega}\triangle\varphi(\nabla\varphi\nabla G_{\lambda}) \,dx +2\sum_{i=1}^{d} \int_{\Omega}\partial_{i}\varphi(\nabla\varphi\partial _{i}\nabla G_{\lambda}) \\ =&\theta+2 \int_{\Omega}\triangle\varphi(\nabla\varphi\nabla G_{\lambda}) \,dx- \int_{\Omega}|\nabla\varphi|^{2}\frac{1}{L-t+\lambda }G_{\lambda}\,dx \\ &{}+2 \int_{\Omega}\biggl(\frac{x}{2(L-t+\lambda)}\nabla\varphi \biggr)^{2}G_{\lambda}\,dx, \end{aligned}$$

where

2.20 $$ \theta= \int_{\partial\Omega}|\nabla\varphi|^{2} \frac{\partial G_{\lambda}}{\partial\nu}\,d \sigma-2 \int_{\partial\Omega} \frac {\partial\varphi}{\partial\nu} (\nabla\varphi\nabla G_{\lambda })\,d\sigma. $$

Meanwhile,

2.21 $$\begin{aligned} \int_{\Omega}\frac{\mu\varphi^{2}}{|x|^{2}}\triangle G_{\lambda }\,dx =&- \int_{\Omega}\nabla\frac{\mu\varphi^{2}}{|x|^{2}}\nabla G_{\lambda }\,dx \\ =&- \int_{\Omega}\frac{2\mu\varphi\nabla\varphi}{|x|^{2}}\nabla G_{\lambda}\,dx+ \int_{\Omega}\frac{2\mu\varphi^{2}x}{|x|^{4}}\nabla G_{\lambda}\,dx \\ =&- \int_{\Omega}\frac{2\mu\varphi\nabla\varphi}{|x|^{2}}\nabla G_{\lambda}\,dx- \frac{1}{L-t+\lambda} \int_{\Omega}\frac{\mu\varphi ^{2}}{|x|^{2}} G_{\lambda}\,dx. \end{aligned}$$

Combining it with (2.18), (2.19), (2.21) indicates

2.22 $$\begin{aligned} D_{\lambda}'(t) =&-2 \int_{\Omega}(\partial_{t}\varphi)^{2} G_{\lambda}\,dx-2 \int _{\Omega}\partial_{t}\varphi\nabla\varphi \frac{-x}{2(L-t+\lambda)} G_{\lambda}\,dx \\ &{}- \theta-2 \int_{\Omega}\triangle\varphi(\nabla\varphi\nabla G_{\lambda}) \,dx-2 \int_{\Omega}\biggl(\frac{x}{2(L-t+\lambda)}\nabla\varphi \biggr)^{2}G_{\lambda}\,dx \\ &{}+ \int_{\Omega}|\nabla\varphi|^{2}\frac{1}{L-t+\lambda}G_{\lambda}\,dx \\ &{}- \int_{\Omega}\frac{2\mu\varphi\nabla\varphi}{|x|^{2}}\nabla G_{\lambda}\,dx- \frac{1}{L-t+\lambda} \int_{\Omega}\frac{\mu\varphi ^{2}}{|x|^{2}} G_{\lambda}\,dx \\ =&-2 \int_{\Omega}(\partial_{t}\varphi)^{2} G_{\lambda}\,dx-4 \int _{\Omega}\partial_{t}\varphi\nabla\varphi \frac{-x}{2(L-t+\lambda)} G_{\lambda}\,dx \\ &{}- 2 \int_{\Omega}\biggl(\frac{x}{2(L-t+\lambda)}\nabla\varphi \biggr)^{2}G_{\lambda}\,dx-\theta+\frac{1}{L-t+\lambda}D_{\lambda}(t) \\ =&-\theta-2 \int_{\Omega}\biggl(\partial_{t} \varphi- \frac{x}{2(L-t+\lambda )}\nabla\varphi\biggr)^{2}G_{\lambda}\,dx+ \frac{1}{L-t+\lambda}D_{\lambda}(t). \end{aligned}$$

Next, we will prove $\theta\geq0$. Since $\varphi=0$ on *∂φ*, it holds that $\nabla\varphi=\frac{\partial\varphi }{\partial\nu}\nu$. For the domain Ω is convex and $0\in\Omega$, we have $x\cdot \nu\geq0$. This, together with (2.7) and (2.20), shows that

$$\begin{aligned} \theta =&-\frac{1}{2(L-t+\lambda)} \int_{\partial\Omega}|\nabla \varphi|^{2} (x\cdot \nu)G_{\lambda}\,d\sigma+\frac{1}{L-t+\lambda } \int_{\partial\Omega} \biggl\vert \frac{\partial\varphi}{\partial\nu} \biggr\vert ^{2} (x\cdot\nu) G_{\lambda}\,d\sigma \\ =&\frac{1}{2(L-t+\lambda)} \int_{\partial\Omega}|\nabla\varphi |^{2} (x\cdot \nu)G_{\lambda}\,d\sigma\geq0. \end{aligned}$$

This completes the proof of this lemma. □

The frequency function $N_{\lambda}(t)$ satisfies the following lemma.

### Lemma 2.4

*For any* $\lambda>0$,

2.23 $$ \lambda N_{\lambda}(L)\leq(L-t+\lambda)N_{\lambda}(t)\leq (L+ \lambda)N_{\lambda}(0),\quad t\in[0,L]. $$

### Proof

By Lemmas 2.2, 2.3, and Remark 2.1, we derive

2.24 $$\begin{aligned} N_{\lambda}'(t) =&\frac{2}{H_{\lambda}^{2}(t)}\bigl\{ D_{\lambda }'(t)H_{\lambda}(t)-H_{\lambda}'(t)D_{\lambda}(t) \bigr\} \\ =&\frac{2}{H_{\lambda}^{2}(t)} \biggl\{ \biggl[-\theta-2 \int_{\Omega}\biggl(\partial _{t} \varphi- \frac{x}{2(L-t+\lambda)}\nabla\varphi\biggr)^{2}G_{\lambda}\,dx+ \frac{1}{L-t+\lambda}D_{\lambda}(t)\biggr] \\ &{}\times\int_{\Omega}\varphi ^{2}G_{\lambda}\,dx+2\biggl( \int_{\Omega}\varphi\biggl(\partial_{t}\varphi-\nabla \varphi\frac {x}{2(L-t+\lambda)} \biggr)G_{\lambda}\,dx\biggr)^{2} \biggr\} \\ \leq&\frac{1}{L-t+\lambda}N_{\lambda}. \end{aligned}$$

The last step is based on the Cauchy–Schwarz inequality. It shows that

2.25 $$\begin{aligned} \bigl[(L-t+\lambda)N_{\lambda}(t) \bigr]'\leq0. \end{aligned}$$

Thus, $(L-t+\lambda)N_{\lambda}(t)$ is a decreasing function, and

2.26 $$ \lambda N_{\lambda}(L)\leq(L-t+\lambda)N_{\lambda}(t)\leq (L+ \lambda)N_{\lambda}(0), \quad t\in[0,L]. $$

This completes the proof of this lemma. □

Letting $m=\sup_{x\in\Omega}\|x\|_{\mathbb{R}^{d}}^{2}$, we have the following.

### Lemma 2.5

*For any* $\lambda>0$,

2.27 $$ \lambda N_{\lambda}(L)\leq\biggl(1+\frac{\lambda}{L}\biggr) \biggl[\frac {m}{L}+2\ln\frac{\int_{\Omega}|\varphi(x,0)|^{2}\,dx}{\int_{\Omega}|\varphi(x,L)|^{2}\,dx} \biggr]. $$

### Proof

We first have

$$ \frac{L}{2}\lambda N_{\lambda}(L)= \int_{0}^{\frac{L}{2}}\lambda N_{\lambda}(L)\,dt. $$

It follows from Lemma 2.4 that

$$ \frac{L}{2}\lambda N_{\lambda}(L)\leq(L+\lambda) \int_{0}^{\frac {L}{2}}N_{\lambda}(t)\,dt=(L+\lambda) \int_{0}^{\frac{L}{2}}\frac {2D_{\lambda}(t)}{H_{\lambda}(t)}\,dt. $$

By Lemma 2.2,

$$ \frac{L}{2}\lambda N_{\lambda}(L)\leq-(L+\lambda) \int_{0}^{\frac {L}{2}}\frac{H_{\lambda}'(t)}{H_{\lambda}(t)}\,dt =(L+\lambda)\ln \frac{H_{\lambda}(0)}{H_{\lambda}(\frac{L}{2})}. $$

Since

$$ \frac{H_{\lambda}(0)}{H_{\lambda}(\frac{L}{2})}\leq\frac{\int_{\Omega}|\varphi(x,0)|^{2}\,dx}{\int_{\Omega}|\varphi(x,\frac{L}{2})|^{2}\,dx} \frac{(\frac{L}{2}+\lambda)^{d/2}}{(L+\lambda)^{d/2}}e^{\frac {m}{4(\frac{L}{2}+\lambda)}} \leq e^{\frac{m}{2L}}\frac{\int_{\Omega}|\varphi(x,0)|^{2}\,dx}{\int _{\Omega}|\varphi(x,\frac{L}{2})|^{2}\,dx}. $$

Therefore,

2.28 $$ \frac{L}{2}\lambda N_{\lambda}(L)\leq(L+\lambda) \biggl[ \frac {m}{2L}+\ln\frac{\int_{\Omega}|\varphi(x,0)|^{2}\,dx}{\int_{\Omega}|\varphi(x,\frac{L}{2})|^{2}\,dx} \biggr]. $$

By direct computation, we obtain

2.29 $$ \frac{d}{dt} \biggl(\frac{1}{2}\|\varphi \|_{L^{2}(\Omega)}^{2} \biggr)=-\|\varphi\|_{H^{1}_{0}(\Omega)}^{2} \leq0. $$

Thus, the solution of (1.1) satisfies that

2.30 $$ \int_{\Omega} \bigl\vert \varphi(x,L) \bigr\vert ^{2} \,dx\leq \int_{\Omega} \biggl\vert \varphi\biggl(x,\frac {L}{2} \biggr) \biggr\vert ^{2}\,dx. $$

We obtain (2.27). This completes the proof of this lemma. □

Since $0\in\omega$, we can get a positive number *r* such that $B_{r}\equiv\{x\in\mathbb{R}^{d} : \|x\|_{\mathbb{R}^{d}}\leq r\} \subset\omega$. The following lemma plays a key role in the proof of the main results.

### Lemma 2.6

*There exists a positive number* $C>1$ *such that*, *for any* $\lambda>0$,

2.31 $$\begin{aligned}& \biggl[1-\frac{8C\lambda}{r^{2}}\biggl(\frac{\lambda}{L}+1 \biggr)\mathcal {K}(L) \biggr] \int_{\Omega} \vert x \vert ^{2} \bigl\vert \varphi(x,L) \bigr\vert ^{2}e^{-\frac { \vert x \vert ^{2}}{4\lambda}}\,dx \\& \quad \leq 8C\lambda \biggl(\frac{\lambda}{L}+1 \biggr)\mathcal{K}(L) \int _{B_{r}} \bigl\vert \varphi(x,L) \bigr\vert ^{2}e^{-\frac{ \vert x \vert ^{2}}{4\lambda}}\,dx, \end{aligned}$$

*where*

2.32 $$ \mathcal{K}(L)\equiv\frac{m}{L}+2\ln\frac{\int_{\Omega}|\varphi (x,0)|^{2}\,dx}{\int_{\Omega}|\varphi(x,L)|^{2}\,dx}+ \frac{d}{2}. $$

### Proof

For any $f(x) \in H_{0}^{1}(\Omega)$, it holds that

2.33 $$ 0\leq \int_{\Omega}\biggl\vert \nabla \biggl(f(x)\exp\biggl(- \frac {|x|^{2}}{8\lambda}\biggr) \biggr) \biggr\vert ^{2}\,dx. $$

By direct computation, we get

2.34 $$ \int_{\Omega}\frac{|x|^{2}}{8\lambda} \bigl\vert f(x) \bigr\vert ^{2}e^{-\frac{|x|^{2}}{4\lambda }}\,dx \leq 2\lambda \int_{\Omega}\bigl\vert \nabla f(x) \bigr\vert ^{2}e^{-\frac {|x|^{2}}{4\lambda}}\,dx+\frac{d}{2} \int_{\Omega}\bigl\vert f(x) \bigr\vert ^{2}e^{-\frac {|x|^{2}}{4\lambda}} \,dx. $$

Recall that, for any $g\in H_{0}^{1}(\Omega)$, the norm $\|g\|_{1}=(\int _{\Omega}(|\nabla g|^{2}-V(x)g^{2})\,dx)^{\frac{1}{2}}$ is equivalent to the standard norm in $H_{0}^{1}(\Omega)$. Thus, there exists a positive number $C>1$ such that

$$ \int_{\Omega}|\nabla g|^{2}\,dx\leq C \int_{\Omega}\bigl(|\nabla g|^{2}-V(x)g^{2} \bigr)\,dx\quad \text{for any } g\in H_{0}^{1}(\Omega). $$

This, combined with (2.34), shows

$$\begin{aligned}& \int_{\Omega} \vert x \vert ^{2} \bigl\vert \varphi(x,L) \bigr\vert ^{2}e^{-\frac{ \vert x \vert ^{2}}{4\lambda }}\,dx \\& \quad \leq 8\lambda \biggl(2\lambda C \int_{\Omega}\biggl[ \bigl\vert \nabla\varphi (x,L) \bigr\vert ^{2}-\frac{\mu \vert \varphi(x,L) \vert ^{2}}{ \vert x \vert ^{2}} \biggr]e^{-\frac { \vert x \vert ^{2}}{4\lambda}}\,dx +\frac{d}{2} \int_{\Omega}\bigl\vert \varphi(x,L) \bigr\vert ^{2}e^{-\frac{ \vert x \vert ^{2}}{4\lambda }}\,dx \biggr) \\& \quad \leq 8\lambda \biggl(\lambda CN_{\lambda}(L) +\frac{d}{2} \biggr) \int_{\Omega}\bigl\vert \varphi(x,L) \bigr\vert ^{2}e^{-\frac { \vert x \vert ^{2}}{4\lambda}}\,dx \\& \quad \leq 8\lambda \biggl(\lambda CN_{\lambda}(L) +\frac{d}{2} \biggr) \biggl( \int_{B_{r}} \bigl\vert \varphi(x,L) \bigr\vert ^{2}e^{-\frac { \vert x \vert ^{2}}{4\lambda}}\,dx+\frac{1}{r^{2}} \int_{\Omega\setminus B_{r}} \vert x \vert ^{2} \bigl\vert \varphi(x,L) \bigr\vert ^{2}e^{-\frac{ \vert x \vert ^{2}}{4\lambda}} \biggr) \\& \quad \leq 8C\lambda\biggl(\frac{\lambda}{L}+1 \biggr)\mathcal{K}(L) \biggl( \int _{B_{r}} \bigl\vert \varphi(x,L) \bigr\vert ^{2}e^{-\frac{ \vert x \vert ^{2}}{4\lambda}}\,dx+\frac {1}{r^{2}} \int_{\Omega\setminus B_{r}} \vert x \vert ^{2} \bigl\vert \varphi(x,L) \bigr\vert ^{2}e^{-\frac { \vert x \vert ^{2}}{4\lambda}} \biggr). \end{aligned}$$

Therefore,

$$\begin{aligned}& \biggl(1-\frac{8C\lambda}{r^{2}}\biggl(\frac{\lambda}{L}+1 \biggr)\mathcal {K}(L) \biggr) \int_{\Omega} \vert x \vert ^{2} \bigl\vert \varphi(x,L) \bigr\vert ^{2}e^{-\frac { \vert x \vert ^{2}}{4\lambda}} \\& \quad \leq 8C\biggl(\frac{\lambda}{L}+1 \biggr)\lambda\mathcal{K}(L) \int _{B_{r}} \bigl\vert \varphi(x,L) \bigr\vert ^{2}e^{-\frac{ \vert x \vert ^{2}}{4\lambda}}\,dx . \end{aligned}$$

This completes the proof of this lemma. □

## Proof of the main result

### Proof

We first prove (1.3). By taking $\lambda>0$ in estimate (2.31) to be such that

3.1 $$ \frac{8C\lambda}{r^{2}} \biggl(\frac{\lambda}{L}+1 \biggr)\mathcal {K}(L)=\frac{1}{2}. $$

By direct computation, we have

$$ \lambda=\frac{1}{2} \biggl(-L+\sqrt{L^{2}+\frac{Lr^{2}}{4C\mathcal {K}(L)}} \biggr). $$

Since $\frac{m}{L}\leq\mathcal{K}(L)$, it follows that

$$\begin{aligned} \frac{1}{\lambda} =&2\frac{L+\sqrt{L^{2}+\frac{Lr^{2}}{4C\mathcal {K}(L)}}}{\frac{Lr^{2}}{4C\mathcal{K}(L)}} \\ =&8C \biggl(L+\sqrt{L^{2}+\frac{Lr^{2}}{4C\mathcal{K}(L)}} \biggr) \frac {1}{Lr^{2}}\mathcal{K}(L) \\ \leq&8C \biggl(2L+\sqrt{\frac{Lr^{2}}{4C\mathcal{K}(L)}} \biggr)\frac {1}{Lr^{2}} \mathcal{K}(L) \\ \leq& \biggl(16+\frac{4r}{\sqrt{Cm}} \biggr)\frac{C}{r^{2}}\mathcal{K}(L). \end{aligned}$$

Therefore, it holds that

3.2 $$\begin{aligned} e^{\frac{m}{4\lambda}} \leq&e^{(4m+r\sqrt{\frac{m}{C}})\frac {C}{r^{2}}\mathcal{K}(L)} \\ \leq&e^{(4m+r\sqrt{\frac{m}{C}})\frac{1}{r^{2}}\frac {d}{2}}e^{(4m+r\sqrt{\frac{m}{C}})\frac{1}{r^{2}}\frac{m}{L}} \biggl(\frac{\int_{\Omega}(|\varphi(x,0)|^{2} )\,dx}{\int _{\Omega}(|\varphi(x,L)|^{2} )\,dx} \biggr)^{2C(4m+r\sqrt {\frac{m}{C}})/ r^{2}}. \end{aligned}$$

By Lemma 2.6, we get

$$ \int_{\Omega}|x|^{2} \bigl\vert \varphi(x,L) \bigr\vert ^{2}e^{-\frac {|x|^{2}}{4\lambda}}\,dx \leq r^{2} \int_{B_{r}} \bigl\vert \varphi(x,L) \bigr\vert ^{2}e^{-\frac {|x|^{2}}{4\lambda}}\,dx. $$

It indicates that

$$\begin{aligned} \int_{\Omega}\bigl\vert \varphi(x,L) \bigr\vert ^{2}e^{-\frac{m}{4\lambda}}\,dx \leq& \int_{\Omega}\bigl\vert \varphi(x,L) \bigr\vert ^{2}e^{-\frac {|x|^{2}}{4\lambda}}\,dx \\ =& \int_{\Omega\setminus B_{r}} \bigl\vert \varphi(x,L) \bigr\vert ^{2}e^{-\frac {|x|^{2}}{4\lambda}}\,dx + \int_{B_{r}} \bigl\vert \varphi(x,L) \bigr\vert ^{2}e^{-\frac{|x|^{2}}{4\lambda }}\,dx \\ \leq&\frac{1}{r^{2}} \int_{\Omega}|x|^{2} \bigl\vert \varphi(x,L) \bigr\vert ^{2}e^{-\frac{|x|^{2}}{4\lambda}}\,dx + \int_{B_{r}} \bigl\vert \varphi(x,L) \bigr\vert ^{2}e^{-\frac{|x|^{2}}{4\lambda }}\,dx \\ \leq&2 \int_{B_{r}} \bigl\vert \varphi(x,L) \bigr\vert ^{2}e^{-\frac {|x|^{2}}{4\lambda}}\,dx \leq2 \int_{B_{r}} \bigl\vert \varphi(x,L) \bigr\vert ^{2} \,dx. \end{aligned}$$

Thus,

3.3 $$\begin{aligned} \int_{\Omega}\bigl\vert \varphi(x,L) \bigr\vert ^{2} \,dx \leq&2e^{\frac{m}{4\lambda}} \int_{B_{r}} \bigl\vert \varphi(x,L) \bigr\vert ^{2} \,dx \\ \leq&2e^{(4m+r\sqrt{\frac{m}{C}})\frac{1}{r^{2}}\frac {d}{2}}e^{(4m+r\sqrt{\frac{m}{C}})\frac{1}{r^{2}}\frac{m}{L}} \biggl(\frac{\int_{\Omega}(|\varphi(x,0)|^{2} )\,dx}{\int _{\Omega}(|\varphi(x,L)|^{2} )\,dx} \biggr)^{2C(4m+r\sqrt {\frac{m}{C}})/ r^{2}} \\ &{}\times\int_{B_{r}} \bigl\vert \varphi(x,L) \bigr\vert ^{2} \,dx. \end{aligned}$$

This shows that

$$ \int_{\Omega}\bigl\vert \varphi(x,L) \bigr\vert ^{2} \,dx\leq Ce^{\frac {C}{r^{2}}}e^{\frac{C}{Lr^{2}}} \biggl(\frac{\int_{\Omega}|\varphi (x,0)|^{2}\,dx}{\int_{\Omega}|\varphi(x,L)|^{2}\,dx} \biggr)^{C/ r^{2}} \int _{B_{r}} \bigl\vert \varphi(x,L) \bigr\vert ^{2}\,dx, $$

which is equivalent to the following inequality:

$$\begin{aligned} \int_{\Omega}\bigl\vert \varphi(x,L) \bigr\vert ^{2} \,dx \leq&Ce^{\frac {C}{L}} \biggl( \int_{\Omega}\bigl|\varphi(x,0)\bigr|^{2}\,dx \biggr)^{\frac{C}{r^{2}+C}} \biggl( \int_{B_{r}} \bigl\vert \varphi(x,L) \bigr\vert ^{2} \,dx \biggr)^{\frac {r^{2}}{r^{2}+C}} \\ \leq&Ce^{\frac{C}{L}} \biggl( \int_{\Omega}\bigl|\varphi_{0}(x)\bigr|^{2}\,dx \biggr)^{\frac{C}{r^{2}+C}} \biggl( \int_{\omega}\bigl\vert \varphi(x,L) \bigr\vert ^{2} \,dx \biggr)^{\frac {r^{2}}{r^{2}+C}}. \end{aligned}$$

Let $\alpha=\frac{r^{2}}{r^{2}+C}$, then the above inequality can be written as

3.4 $$ \int_{\Omega}\bigl\vert \varphi(x,L) \bigr\vert ^{2} \,dx \leq Ce^{\frac {C}{L}} \biggl( \int_{\Omega}\bigl|\varphi_{0}(x)\bigr|^{2}\,dx \biggr)^{1-\alpha} \biggl( \int_{\omega}\bigl\vert \varphi(x,L) \bigr\vert ^{2} \,dx \biggr)^{\alpha}. $$

Conclusion (1.3) then follows.

In order to prove (1.4), we will prove the following estimate:

3.5 $$ \bigl\Vert \varphi(x,0) \bigr\Vert _{H^{-1}(\Omega)}^{2} \leq\exp \biggl( CL\frac{ \Vert \varphi(x,0) \Vert _{H^{-1}(\Omega)}^{2}}{ \Vert \varphi(x,0) \Vert _{H^{-1}(\Omega )}^{2}} \biggr) \bigl\Vert \varphi(x,L) \bigr\Vert _{H^{-1}(\Omega)}^{2}. $$

We define a function $\Phi(t)$ as follows:

$$ \Phi(t)=\frac{\|\varphi(x,t)\|_{L^{2}(\Omega)}^{2}}{\|\varphi(x,t)\| _{H^{-1}(\Omega)}^{2}}. $$

By direct computation, we obtain

3.6 $$ \frac{d}{dt} \biggl(\frac{1}{2}\|\varphi \|_{H^{-1}(\Omega)}^{2} \biggr)=-\|\varphi\|_{L^{2}(\Omega)}^{2}. $$

This, together with (2.4) and (2.29), indicates

$$\begin{aligned} \frac{d}{dt}\Phi(t) =&\frac{ (\|\varphi\|_{L^{2}(\Omega )}^{2} )' (\|\varphi\|_{H^{-1}(\Omega)}^{2} )- (\| \varphi\|_{L^{2}(\Omega)}^{2} ) (\|\varphi\|_{H^{-1}(\Omega )}^{2} )'}{ (\|\varphi\|_{H^{-1}(\Omega)}^{2} )^{2}} \\ =&\frac{2}{ (\|\varphi\|_{H^{-1}(\Omega)}^{2} )^{2}} \bigl\{ -\|\varphi\|_{H^{1}_{0}(\Omega)}^{2}\|\varphi \|_{H^{-1}(\Omega)}^{2} +\|\varphi\|_{L^{2}(\Omega)}^{4} \bigr\} \leq0. \end{aligned}$$

Thus, $\Phi(t)$ is a decreasing function, and

$$ \Phi(L)\leq \Phi(0). $$

It follows from (2.29) and (3.6) that

3.7 $$\begin{aligned} 0 =&\frac{1}{2}\frac{d}{dt} \bigl(\|\varphi\|_{H^{-1(\Omega )}}^{2} \bigr)+\|\varphi\|_{L^{2}(\Omega)}^{2} \\ \leq&\frac{1}{2}\frac{d}{dt} \bigl(\|\varphi\|_{H^{-1}(\Omega )}^{2} \bigr)+\Phi(0)\|\varphi\|_{H^{-1}(\Omega)}^{2}. \end{aligned}$$

Integrating (3.7) on $(0,L)$, we get the desired estimate

$$ \bigl\Vert \varphi(x,0) \bigr\Vert _{H^{-1}(\Omega)}^{2}\leq e^{2\Phi(0)L} \bigl\Vert \varphi (x,L) \bigr\Vert _{H^{-1}(\Omega)}^{2}. $$

With the aid of (3.5), we can get (1.4). This completes the proof. □

### Corollary 3.1

*Suppose that* *ω* *is a non*-*empty open subset of* Ω, $0\in\omega$, *and* $\varphi_{0}\in L^{2}(\Omega)$. *Then there exist two positive numbers* $\alpha=\alpha(\Omega,\omega)$, $C=C(\Omega,\omega)$ *such that*, *for each* $L>0$ *and* $\tilde{\Omega }\Subset\Omega$,

3.8 $$ \int_{\tilde{\Omega}} \bigl\vert \varphi(x,L) \bigr\vert ^{2} \,dx\leq Ce^{\frac {2C}{L}}L^{\alpha-1}\bigl( \bigl\Vert \varphi(x,s) \bigr\Vert _{{L^{2}(\Omega\times (0,L))}}\bigr)^{1-\alpha}\biggl( \int_{\omega}\bigl\vert \varphi(x,L) \bigr\vert ^{2} \,dx\biggr)^{\alpha}. $$

### Proof

For any $s\in[0,\frac{L}{2}]$, we take $z(x,t)=\varphi(x, t+s)$, where $t\in[0, L-s]$, $x\in\Omega$. Then $z(t,x)$ satisfies the following equation:

$$ \textstyle\begin{cases} \partial_{t}z(x,t)-\triangle z(x,t)-V(x)z(x,t)=0 & \text{in } \Omega\times(0,L-s], \\ z(x,t)=0 & \text{on } \partial\Omega\times(0,L-s], \\ z(x,0)=\varphi(s,x) & \text{in }\Omega. \end{cases} $$

By the same argument as that in the proof of Theorem 1.1, we also get

$$ \int_{\Omega}\bigl\vert z(x,L-s) \bigr\vert ^{2}\,dx \leq Ce^{\frac{C}{L-s}}\biggl( \int_{\Omega}\bigl\vert z(x,0) \bigr\vert ^{2}\,dx \biggr)^{1-\alpha}\biggl( \int_{\omega}\bigl\vert z(x,L-s) \bigr\vert ^{2}\,dx \biggr)^{\alpha}, $$

where the constant *C* is a positive constant only depending on Ω and *ω*.

Thus,

$$ \int_{\Omega}\bigl\vert \varphi(x,L) \bigr\vert ^{2} \,dx\leq Ce^{\frac{C}{L-s}}\biggl( \int_{\Omega}\bigl\vert \varphi(x,s) \bigr\vert ^{2} \,dx\biggr)^{1-\alpha}\biggl( \int_{\omega}\bigl\vert \varphi (x,L) \bigr\vert ^{2}\,dx\biggr)^{\alpha}. $$

Then we have

3.9 $$\begin{aligned} \frac{L}{2} \int_{\Omega}\bigl\vert \varphi(x,L) \bigr\vert ^{2} \,dx =& \int_{0}^{\frac{L}{2}} \int_{\Omega}\bigl\vert \varphi(x,L) \bigr\vert ^{2} \,dx\,ds \\ \leq& \int_{0}^{\frac{L}{2}}Ce^{\frac{C}{L-s}}\biggl( \int_{\Omega}\bigl\vert \varphi (x,s) \bigr\vert ^{2}\,dx\biggr)^{1-\alpha}\biggl( \int_{\omega}\bigl\vert \varphi(x,L) \bigr\vert ^{2} \,dx\biggr)^{\alpha }\,ds \\ \leq& Ce^{\frac{2C}{L}}\biggl( \int_{\omega}\bigl\vert \varphi(x,L) \bigr\vert ^{2} \,dx\biggr)^{\alpha } \int_{0}^{\frac{L}{2}}\biggl( \int_{\Omega}\bigl\vert \varphi(x,s) \bigr\vert ^{2} \,dx\biggr)^{1-\alpha}\,ds \\ \leq& Ce^{\frac{2C}{L}}\biggl(\frac{L}{2}\biggr)^{\alpha}\biggl( \int_{\omega}\bigl\vert \varphi(x,L) \bigr\vert ^{2} \,dx\biggr)^{\alpha}\bigl( \bigl\Vert \varphi(x,s) \bigr\Vert _{{L^{2}(\Omega\times (0,\frac{L}{2}))}}\bigr)^{1-\alpha}. \end{aligned}$$

The last step is obtained by Hölder’s inequality. Therefore, we can get (3.8). This completes the proof. □
